# Circulating tumour cells are associated with increased risk of venous thromboembolism in metastatic breast cancer patients

**DOI:** 10.1038/sj.bjc.6605413

**Published:** 2009-11-03

**Authors:** M Mego, U De Giorgi, K Broglio, S Dawood, V Valero, E Andreopoulou, B Handy, J M Reuben, M Cristofanilli

**Affiliations:** 1 Department of Hematopathology, University of Texas, MD Anderson Cancer Center, Houston, TX, USA; 2 Department of Breast Medical Oncology, Morgan Welch Inflammatory Breast Cancer Research Program and Clinic, University of Texas, MD Anderson Cancer Center, Houston, TX, USA; 3 Department of Medical Oncology, Comenius University, School of Medicine, Bratislava, Slovakia; 4 Department of Biostatistics, University of Texas, MD Anderson Cancer Center, Houston, TX, USA

**Keywords:** circulating tumour cells, venous thromboembolism

## Abstract

**Background:**

Cancer is a risk factor for venous thromboembolism (VTE). Circulating tumour cells (CTCs) are an independent predictor of survival in metastatic breast cancer (MBC) patients. The aim of this study was to test the hypothesis that CTCs are associated with the risk of VTE in MBC patients.

**Methods:**

This retrospective study included 290 MBC patients treated in the MD Anderson Cancer Center from January 2004 to December 2007. Circulating tumour cells were detected and enumerated using the CellSearch system before starting new lines of therapy.

**Results:**

At a median follow-up of 12.5 months, 25 patients experienced VTE and 53 patients died without experiencing thrombosis. Cumulative incidence of thrombosis at 12 months was 8.5% (95% confidence interval (CI)=5.5%, 12.4%). Patients with CTCs ⩾1 and ⩾5 had a higher incidence of VTE compared with patients with 0 and <5 CTCs (12-month estimate, 11.7 and 11.6% *vs* 3 and 6.6%; *P*=0.006 and *P*=0.076, respectively). In the multivariate model, patients with CTCs⩾1 had a hazard ratio of VTE of 5.29 (95% CI=1.58, 17.7, *P*=0.007) compared with patients with no CTCs.

**Conclusion:**

These results suggest that CTCs in MBC patients are associated with increased risk of VTE. These patients should be followed up more closely for the risk of VTE.

Cancer is a well-recognised risk factor for venous thromboembolism (VTE). It has been shown that 5–10% of all cancer patients will develop VTE during the course of the disease (Silverstein *et al*, 1998). Evidence suggests that the absolute risk depends on the tumour type, the stage or extent of the cancer, and treatment with antineoplastic agents (Silverstein *et al*, 1998).

Venous thromboembolism following breast cancer chemotherapy is common. In early breast cancer, VTE occurs in 5–10% of patients receiving chemotherapy (Weiss *et al*, 1981; Levine *et al*, 1988; von Tempelhoff *et al*, 1996), and it rises up to 18% in advanced breast cancer with 9% mortality (Goodnough *et al*, 1984; Kirwan *et al*, 2008).

Circulating tumour cells (CTCs) are an independent predictor of progression-free survival (PFS) and overall survival (OS) in patients with metastatic breast cancer (MBC) (Cristofanilli *et al*, 2004). Superior survival among patients with <5 CTCs was observed regardless of histology, hormone receptor and HER2/neu status, sites of first metastases, or whether the patient had relapse or *de novo* metastatic disease (Cristofanilli *et al*, 2004; Dawood *et al*, 2008).

Increased CTC count and VTE are poor prognostic factors in MBC and are linked to inferior survival. In this retrospective study, we tested the hypothesis that CTCs are associated with the risk of VTE in MBC patients.

## Patients and methods

### Study patients

This study was conducted using the MD Anderson Cancer Center medical records database. The retrospective study was approved by the institutional review board and a waiver of consent form was granted. A population of consecutive MBC patients with at least one measurement of CTC before starting a new line of therapy from January 2004 to December 2007 was eligible. In addition, patients were not excluded on the basis of whether they underwent treatment with any particular form of chemotherapy, hormonal therapy, or biological therapy. Patients on prophylactic or therapeutic anticoagulation therapy including warfarin 1 mg per day or equivalent for port-a-catheter thromboprophylaxis, low molecular weight heparin, or unfractionated heparin were excluded from the analysis. Patients with concurrent malignancy other than non-melanoma skin cancer in the previous 5 years were excluded as well. In all, patient data regarding age, tumour histology, hormone receptor status, HER2 status, type and number of metastatic sites, systemic therapy, history of VTE, comorbidities (hypertension, diabetes mellitus), and concomitant therapy were also recorded and compared with risk of VTE.

### Definition of the events

All venous thrombosis and/or pulmonary embolism in the presence of unequivocal medical documentation were classified as events. A patient was considered to have had a VTE if the event was clinically apparent and confirmed by diagnostic studies. Cases of superficial phlebitis and cases of secondary thrombosis attributed to superior vena cava syndrome and/or bulky abdominal lymphadenopathy were not classified as events and were excluded from the analysis.

### Detection of CTCs in peripheral blood

The CellSearch system (Veridex Corporation, Warren, NJ, USA) was used to detect CTCs in 7.5 ml of whole peripheral blood. Samples were subject to enrichment with anti-EpCAM-coated beads. Circulating tumour cells were defined as nucleated cells lacking CD45 but expressing cytokeratines 8, 18, or 19.

### Statistical analysis

Baseline CTCs were defined as the earliest CTC measurement taken before the start of a new line of therapy. Time to thrombosis was calculated from the date of baseline CTC assessment to the date of thrombosis or last follow-up. We calculated the cumulative incidence of thrombosis according to the method previously described (Gray, 1988). We considered baseline CTCs as a continuous measurement, dichotomised at 1 and at 5. The cutoff at 1 was chosen because it has been investigated in other settings such as primary breast cancer (Cristofanilli *et al*, 2004; Lang *et al*, 2009). The cutoff at 5 has been established as prognostic for PFS and OS for MBC patients in other studies.

Analyses were repeated considering patients who died before experiencing a thrombosis as censored at their date of death and estimating survival from thrombosis according to the Kaplan–Meier method. Results were similar. Therefore, we used Cox proportional hazards models both to assess CTCs as continuous measurements and to determine the association between CTCs and thrombosis after adjustment for other patient and disease characteristics.

Analyses were conducted in R2.4.0 with the contributed package, cmprsk (Gray, 2004; R Development Core Team, 2006). *P*-values <0.05 were considered statistically significant.

## Results

We identified 290 patients who satisfied the study eligibility criteria and were included in this analysis. Patient characteristics are shown in Table 1.

A total of 25 patients experienced a thrombosis and 53 patients died without experiencing a thrombosis. Estimates of the cumulative incidence of thrombosis are shown in Table 2. Among all patients, the cumulative incidence of thrombosis at 12 months was 8.5% (95% confidence interval (CI)=5.5%, 12.4%). There was no association between baseline CTCs and thrombosis when baseline CTCs were considered as continuous in a univariate Cox proportional hazards model (hazards ratio (HR)=1.0, 95% CI=0.994, 1.00, *P*=0.73). When baseline CTCs were considered dichotomised at 1, patients with CTCs⩾1 had four times higher incidence of thrombosis compared with patients with CTC=0 (12-month estimate 3.0 *vs* 11.7%, *P*=0.006). Patients with CTCs⩾1 have inferior survival compared with patients with CTC=0 (HR=0.54, 95% CI=0.33–0.89, *P*=0.03). When patients were considered grouped according to CTCs⩾5 *vs* CTCs<5, patients with fewer CTCs had a lower incidence of thrombosis compared with patients with more CTCs; however, statistical significance was not attained (6.6 *vs* 11.6%, *P*=0.076).

We considered the baseline CTC measurement dichotomised as 0 *vs* 1 or more in a multivariable Cox proportional hazards model to determine whether the association with thrombosis persisted after adjustment for other characteristics After adjustment for these other terms, having at least one CTC was associated with 5.29 times the risk of thrombosis compared with patients with no CTC (95% CI=1.58, 17.7, *P*=0.007) (Table 3).

## Discussion

This large single centre retrospective study showed that CTCs are associated with increased risk of VTE in MBC patients. The risk is increased in patients with CTCs⩾1 before starting new line of therapy. Observed cumulative 12-month incidence of VTE in our patients was 8.5%, which is in concordance with data from literature. (Ottinger *et al*, 1995; Baron *et al*, 1998). We confirmed that the presence of visceral metastases, increased number of metastases, and subsequent lines of therapy are associated with increased risk of VTE. These factors mainly reflect advanced disease, with higher incidence of VTE at all.

In a prospective, multicentre study, the number of CTCs before chemotherapy was an independent predictor of PFS and OS in MBC patients. Although the threshold of 5 CTCs per 7.5 ml of blood has been shown to be prognostic for survival (Cristofanilli *et al*, 2004), in our study, any detectable CTCs were associated with increased risk for VTE as well as with increased risk of death. We also observed that MBC patients with CTCs⩾5 have a doubled risk of VTE compared with patients with CTCs<5; however, this difference did not reach statistical significance.

There are several mechanisms that may explain this association (CTC and VTE). Increased CTC count is a marker of more aggressive disease with increased risk of VTE (Cristofanilli *et al*, 2004). Circulating tumour cells could be directly involved in coagulation activation as well. It is supposed that the direct toxic effect of anticancer treatment on cancer cells may lead to an increase in CTC fragments or microparticles with procoagulant activity (Dvorak *et al*, 1983). Circulating tumour cells could be involved in the activation of coagulation through the expression and release of tissue factors (TFs) (Davila *et al*, 2008). It was shown that TFs are overexpressed in cells with cancer stem cell phenotype (Milsom *et al*, 2007). At least the subgroups of CTCs are potential cancer stem cells (Reuben *et al*, 2007); therefore, CTCs could be an important source of TFs and could be involved directly in coagulation activation.

The main limitation of this trial is the retrospective nature of analysis. Therefore, the study results are only hypothesis generating. Sample size, heterogeneous patient population, and heterogenity of therapy might affect the study results. On the other hand, the majority of patients in our analysis were treated according to daily clinical practice, which might increase the generalisability of the results.

To our knowledge, this is the first study to assess the prognostic value of CTCs on the risk of VTE. Patients with MBC and any detectable CTCs are at increased risk for VTE. These patients should be followed more closely for the risk of VTE. Further research in this field is warranted, with prospective assessment of coagulation status and its correlation with CTC count and clinical outcome.

## Figures and Tables

**Table 1:** Patient characteristics (*n*=290)

	***N***	**Percent**
Median age; years (range)	54 (23–84)	
Median baseline CTC (range)	2 (0–1780)	
		
*Line of therapy*		
1	123	42.41
2 or more	167	57.59
		
*Estrogen and progesteron receptor*		
Positive for both	192	66.21
Negative for either	98	33.79
		
*HER2/neu amplified*		
No	227	78.28
Yes	62	21.38
Unknown	1	0.34
		
*Inflammatory breast cancer*		
No	222	76.55
Yes	68	23.45
		
*Visceral metastasis*		
No	109	37.59
Yes	181	62.41
		
*Bone metastasis*		
No	88	30.34
Yes	202	69.66
		
*Number of sites of metastasis*		
1	100	34.48
2	89	30.69
3	55	18.97
⩾4	46	15.86
		
*Chemotherapy*		
No	36	12.41
Yes	254	87.59
Bevacizumab-based therapy	60	20.69
		
*Hormonal therapy*		
No	164	56.55
Yes	126	43.45
		
*Erythropoietin-stimulating agents*		
No	256	88.28
Yes	34	11.72
		
*Port-a-catheter and/or central venous device*		
No	211	72.76
Yes	79	27.24
		
*Arterial hypertension*		
No	188	64.83
Yes	102	35.17
		
*Diabetes mellitus*		
No	255	87.93
Yes	35	12.07

Abbreviation: CTC=circulating tumour cells.

**Table 2:** Estimates of the cumulative incidence of thrombosis

	***N***	***No of* thrombosis events**	**12-Month estimate (percent)**	**95% Confidence interval**	***P*-value**
All	290	25	8.5	(5.5, 12.4)	—
					
*Baseline CTC*					
0	108	3	3.0	(0.8, 7.9)	
⩾1	182	22	11.7	(7.3, 17.3)	0.006
					
*Baseline CTC*					
<5	177	11	6.6	(3.3, 11.4)	
⩾5	113	14	11.6	(6.3, 18.6)	0.076
					
*Age (years)*					
<50	100	10	8.9	(4.1, 15.9)	
>50	190	15	8.3	(4.7, 13.2)	0.552
					
*Line of therapy*					
1	123	6	3.3	(1.1, 7.8)	
⩾2	167	19	12.8	(7.8, 19.1)	0.027
					
*Estrogen and progesteron receptor*					
Positive for both	192	14	6.4	(3.4, 10.8)	
Negative for either	98	11	12.8	(6.7, 21.0)	0.188
					
*HER2/neu amplified*					
No	227	20	8.4	(5.1, 12.8)	
Yes	62	5	8.8	(3.2, 18.1)	0.900
					
*Inflammatory breast cancer*					
No	222	17	7.9	(4.7, 12.1)	
Yes	68	8	11.1	(4.3, 21.3)	0.234
					
*Visceral metastasis*					
No	109	2	2.0	(0.4, 6.3)	
Yes	181	23	12.3	(7.8, 18.0)	0.002
					
*Bone metastasis*					
No	88	6	8.6	(3.4, 16.9)	
Yes	202	19	8.6	(5.1, 13.2)	0.547
					
*Number of sites of metastasis*					
1	100	3	3.53	(0.9, 9.2)	
2 or 3	144	11	7.69	(3.9, 13.2)	
⩾4	46	11	21.07	(10.2, 34.5)	0.002
					
*Chemotherapy*					
No	36	3	5.6	(1.0, 16.5)	
Yes	254	22	8.8	(5.6, 13.0)	0.934
					
*Bevacizumab-based therapy*					
No	230	20	8.1	(4.9, 12.4)	
Yes	60	5	10.0	(3.5, 20.5)	0.999
					
*Hormonal therapy*					
No	164	19	11.9	(7.2, 18.0)	
Yes	126	6	4.2	(1.6, 8.9)	0.037
					
*Tamoxifen*					
No	101	10	10.4	(5.3, 17.5)	
Yes	38	1	0.0	—	0.177
					
*Aromatase inhibitors*					
No	194	22	11.2	(7.0, 16.6)	
Yes	96	3	3.2	(0.9, 8.3)	0.016
					
*Erythropoetin-stimulating agents*					
No	256	24	9.3	(5.9, 13.6)	
Yes	34	1	2.9	(0.2, 13.2)	0.150
					
*Port-a-catheter and/or central venous device*					
No	211	17	7.9	(4.6, 12.4)	
Yes	79	8	9.8	(4.2, 18.1)	0.747
					
*History of DVT/PE*					
No	274	23	8.0	(5.1, 11.9)	
Yes	16	2	14.6	(2.0, 38.7)	0.672
					
*Arterial hypertension*					
No	188	19	9.1	(5.4, 13.9)	
Yes	102	6	7.5	(3.0, 14.8)	0.242
					
*Diabetes mellitus*					
No	255	22	8.5	(5.3, 12.6)	
Yes	35	3	8.9	(2.2, 21.6)	0.924

Abbreviations: DVT=deep vein thrombosis; PE= pulmonary embolism, CTC=circulating tumour cells.

**Table 3:** Cox proportional hazards model

	**Hazard ratio**	**Lower 95% CI**	**Upper 95% CI**	***P*-value**
Baseline CTC (⩾1 *vs* 0)	5.29	1.58	17.70	0.007
Line of therapy (⩾2 *vs* 1)	2.53	1.00	6.40	0.049
Number of metastatic sites (2 or 3 *vs* 1)	2.81	0.78	10.10	0.110
Number of metastatic sites (4 or 5 *vs* 1)	8.08	2.24	29.10	0.001

Abbreviations: CI= confidence interval; CTC=circulating tumour cells.
